# Correlation of Infarct Volume, Collateral Status, and Reperfusion with Clinical Outcome Following Mechanical Thrombectomy in Acute Ischemic Stroke

**DOI:** 10.7759/cureus.98337

**Published:** 2025-12-02

**Authors:** Priyanka Cheekatla, Bijjiga Siphora Krupalini, Akudari Vidyasagar, Repalle Prasanna, Repalle Prashanthi, Buddhapuram Pranav Krishna, Misri Zulkifli

**Affiliations:** 1 Department of Neurology, KMC Hospital, Mangalore, IND; 2 Department of Neurology, Aster Medcity, Kochi, IND; 3 Department of Internal Medicine, Sudheeksha Multispeciality Hospital, Nalgonda, IND; 4 Department of Radiodiagnosis, Bhaskar Medical College, Moinabad, IND; 5 Department of Pulmonology, Employees' State Insurance (ESI) Corporation Medical College and Hospital, Hyderabad, IND; 6 Department of Orthopaedics, Bhaskar Medical College, Moinabad, IND; 7 Department of Neurology, Kasturba Medical College Hospital, Mangalore, IND

**Keywords:** collaterals, diffusion-weighted mri, infarct core volume, mechanical thrombectomy, mtici, prognosis, symptomatic intracranial haemorrhage

## Abstract

Background: Mechanical thrombectomy (MT) has widened eligibility beyond rigid time windows, yet outcomes still vary after angiographic success. Imaging-based core size, collateral status, and reperfusion quality may refine prognosis, while haemorrhagic complications can negate gains.

Objective: To evaluate whether infarct core volume, collateral grade, and angiographic reperfusion independently associate with 90-day outcome after MT, to quantify the impact of symptomatic intracranial haemorrhage (sICH), and to identify an anterior-circulation core-volume threshold predictive of poor prognosis.

Methods: Single-centre, prospective cohort of adults with acute ischemic stroke (AIS) undergoing MT within 24 h (n = 41). Baseline core volume was measured on diffusion-weighted magnetic resonance imaging (MRI) (diffusion weighted image, DWI) using the ABC/2 method. Baseline core volume on DWI (ABC/2), entered per 10 mL, collateral grade (American Society of Interventional and Therapeutic Neuroradiology/Society of Interventional Radiology (ASITN/SIR) for anterior; Basilar Artery on Computed Tomography Angiography (BATMAN) score ≥7 = good for posterior), and reperfusion modified Thrombolysis in Cerebral Infarction (mTICI) were evaluated. sICH was defined per European Cooperative Acute Stroke Study (ECASS) III. Reperfusion was scored by mTICI. Functional outcome at 90 days was dichotomised as good (modified Rankin Scale (mRS) ≤ 2 versus poor (mRS ≥ 3). Multivariable logistic regression tested independent associations. Receiver operating characteristic (ROC) analysis assessed the discriminatory performance of a pragmatic anterior-circulation core threshold.

Results: Baseline DWI core volume, higher mTICI grade, and sICH were independent predictors of 90-day outcome; collateral scores and time metrics were not retained after adjustment. A ~50 mL anterior-circulation core threshold demonstrated high discrimination for poor prognosis (area under the curve (AUC) = 0.91). sICH occurred in 34.1% (14/41) and strongly tracked with poor functional recovery. Reperfusion quality remained positively associated with independence at 90 days despite adjustment for core and sICH.

Conclusions: Outcome after MT is chiefly determined by tissue burden at baseline, completeness of reperfusion, and avoidance of sICH. A ~50 mL DWI core threshold is a practical risk flag for counselling and audit, but should not be an absolute barrier to treatment. Given the small, single-centre sample and an unusually high sICH rate, these findings are exploratory and have limited generalisability; external validation in larger, harmonised cohorts is required.

## Introduction

Mechanical thrombectomy (MT) has changed acute stroke care for large-vessel occlusion: many patients who would otherwise remain disabled can now recover [1,2]. Indications have broadened from early-window anterior circulation strokes to late or unknown onset, larger infarcts, and basilar artery occlusion, supported by better devices, faster systems, and imaging-guided selection [1,3]. Still, outcomes differ even after a technically successful procedure. Patients of similar age, baseline National Institutes of Health Stroke Scale (NIHSS), and treatment times can have very different 90-day modified Rankin Scale (mRS) results. The central questions are straightforward: how much brain was already infarcted, how well collateral flow supported threatened tissue, and how completely blood flow was restored [3,4]?

Recent randomized and guideline signals have cautiously walked into the “large-core” space and the late window, reframing selection around tissue and physiology rather than clock time [1,4]. A trial-only meta-analysis across modern large-core randomized controlled trials (RCTs) shows higher chances of functional independence with endovascular therapy (EVT) despite an expected increase in intracranial haemorrhage, and without a mortality penalty [5]. Observational data reinforce that benefit is not linear across the core spectrum: in the Infrastructure for Spatial Information in Europe (INSPIRE) registry, EVT advantage was prominent for core volumes of 70-100 mL but disappeared and even hinted harm at ≥100 mL [6]. An earlier synthesis suggested thrombectomy can help patients selected by low Alberta Stroke Program Early CT Score (ASPECTS), but evidence becomes equivocal when selection relies purely on quantitative core volume thresholds, reflecting heterogeneous definitions and methods [7]. Editorial commentary has therefore urged patient-level meta-analyses to examine whether core size truly modifies treatment effect or whether current subgroup signals are artefacts of limited power and mixed imaging criteria [8]. Meanwhile, pragmatic randomized programs continue to test EVT against best medical care under real-world constraints and with clear imaging rules, including PROBE-style designs and public-system trials that aim to translate efficacy into policy [9,10].

Collateral circulation is the second pillar. It governs the tempo of infarct growth, modulates hemorrhagic risk after reperfusion, and consistently tracks with 90-day function across thrombolysis and thrombectomy cohorts [11]. Large registries confirm that better angiographic collaterals, as predicted by the American Society of Interventional and Therapeutic Neuroradiology/Society of Interventional Radiology (ASITN/SIR); three to four predict independence at follow-up, while also showing that successful reperfusion helps across the board; the penalty for time is simply steeper when collaterals are poor [12]. Even in very old patients, those ≥90 years, MRI surrogates such as Fluid-Attenuated Inversion Recovery (FLAIR) vascular hyperintensities (FVH) vascular hyperintensity scores capture meaningful differences in survival and recovery, suggesting that simple collateral markers can retain prognostic value in frail groups where treatment margins are narrow [13]. The venous side of the circuit has stepped forward as well: multiphase computed tomography angiography (CTA)-derived venous-outflow scores (e.g., Cortical Vein Opacification Score; COVES) [14] forecast both the likelihood of excellent reperfusion and downstream outcomes, offering a practical selector when perfusion software is unavailable or would slow care [15].

Reperfusion quality is the third leg of the stool, less glamorous in concept than “who to treat,” but decisive in practice. Achieving expanded Thrombolysis in Cerebral Infarction (eTICI) 2c/3 [16], rather than settling for 2b, associates with sharper functional gains at 90 days [17]. Tissue-level collateral status, measurable by the hypoperfusion intensity ratio (HIR) [18], appears to modify that return on investment: in patients with poor collaterals (higher HIR), pushing toward near-complete reperfusion buys a larger functional dividend; in those with strong collaterals (HIR <0.3), the marginal benefit of 2c/3 over 2b compresses [17]. Real-world registries remind us that the route to reperfusion is not harmless; subarachnoid haemorrhage/perforation, distal embolization, and symptomatic intracranial haemorrhage (sICH) [19] are infrequent but impactful, and each complication meaningfully worsens outcomes [20]. Post-procedure care remains part of the causal chain: blood-pressure targets, early imaging to detect haemorrhage or oedema, and vigilance for re-occlusion can tilt trajectories even after the catheter has left the body [4,21]. Technique debates direct MT versus intravenous (IV)-bridge have settled into a pragmatic truce: functional outcomes look broadly similar, with trade-offs between angiographic success and haemorrhage profiles, reinforcing that patient biology and reperfusion quality may overshadow alteplase exposure in many scenarios [22].

What follows is a practical synthesis: a small, coherent set of imaging biomarkers captures the biology (core already lost), the hemodynamics (collateral supply), and the craft (reperfusion grade). Infarct volume derived from diffusion-weighted magnetic resonance imaging (MRI) (DWI) core, CT-perfusion rCBF <30%, or ASPECTS [23] anchors baseline injury and the ceiling for recovery. Collaterals graded on multiphase CTA, encoded on FLAIR as vascular hyperintensity, or inferred from perfusion surrogates like HIR, contextualize both infarct kinetics and haemorrhage risk [11,24]. Reperfusion quality codified by modified Thrombolysis in Cerebral Infarction (mTICI)/eTICI summarizes the procedural endpoint that teams can standardize and audit. Beyond the baseline scan, early post-MT imaging holds independent value: 24-hour DWI lesion size and infarct growth strongly and inversely track with 90-day independence and can refine monitoring intensity, escalation decisions, and counseling [25]. Together, these measures move selection beyond the clock and make prognostication less guesswork and more quantification.

Guidelines have evolved to support this tissue-first posture. For late-window candidates, clinical-imaging mismatch remains the anchor; when advanced imaging would introduce harmful delay, non-contrast computed tomography (NCCT)-based pathways are acceptable, provided systems are disciplined and teams are trained [4]. Contemporary practice standards integrating large-core RCTs emphasize consistent adjudication, imaging training, and core-lab support, aligning day-to-day decisions with the best available evidence and reminding clinicians that age or pre-stroke disability alone should not exclude patients when other criteria point toward benefit [3]. Parallel design papers and national trials ensure these advances are tested where resources are stretched, aiming to convert evidence into equitable access and cost-effective policy [9,10].

This study is positioned at the junction of tissue burden, collateral physiology, and procedural success. By quantifying infarct volume, grading collaterals with validated scales, and capturing reperfusion quality with standardized angiographic metrics, it seeks to explain why similar “technical wins” can yield different human outcomes. The intention is practical: sharpen prognostication for patients and families, identify subgroups that demand more aggressive pursuit of near-complete reperfusion, and specify thresholds that might trigger caution when the core is already too large for meaningful recovery [1,2,6].

This study aims to evaluate whether infarct volume, collateral circulation, and reperfusion grade independently predict 90-day functional outcome after mechanical thrombectomy in acute ischemic stroke, and to identify an infarct-volume threshold that predicts poor prognosis.

## Materials and methods

Study design and setting

Prospective observational cohort of 41 consecutive adults with acute ischemic stroke (AIS) undergoing mechanical thrombectomy (MT) within 24 hours of last-known-well or witnessed onset. The study was structured to test whether baseline infarct volume, collateral status, and angiographic reperfusion independently predict 90-day functional outcome, and to explore a pragmatic infarct-volume threshold associated with poor prognosis. Methods followed routine clinical workflows at a tertiary stroke centre and standard endovascular reporting practices. This study was conducted at the Department of Neurology, Kasturba Medical College (KMC) Hospital, Ambedkar Circle, Mangalore, with approval of IECKMCMLR-12/2022/492. This study was conducted between January and June 2023.

Participants

Inclusion criteria: age ≥18 years; anterior or posterior circulation large-vessel occlusion treated by MT ≤24 hours; baseline DWI adequate for core estimation; and 90-day outcome ascertainment. Exclusion criteria for analysis were the absence of evaluable baseline DWI or missing 90-day modified Rankin Scale (mRS) [26] data. Demographics, vascular risk factors, NIHSS [27], and key time points were abstracted from clinical records. The centre’s thrombectomy pathway required pre-procedural MRI-DWI to quantify core volume; patients assessed solely with NCCT/CTA were not considered for MT during the study period.

Imaging acquisition and measurements

Infarct volume. Baseline DWI defined ischemic core. Volumes (mL) were estimated using the ABC/2 method [28]: A = largest lesion diameter on the axial slice with greatest involvement; B = orthogonal diameter on the same slice; C = slice thickness × number of involved slices; volume = (A×B×C)/2. Multifocal lesions were summed. Collateral status. Collaterals were graded by vascular territory: ASITN/SIR (0-4) [29] for the anterior circulation and Basilar Artery on Computed Tomography Angiography (BATMAN) [30] score for the posterior circulation. For analyses, collateral status was handled as ordinal grades and dichotomised a priori into “good” vs “poor” per each scale’s conventional thresholds. Reperfusion assessment. Post-MT angiographic reperfusion was graded by mTICI [31]. Primary analyses contrasted mTICI 2b-3 versus <2b; exploratory analyses examined near-complete/complete reperfusion (eTICI 2c/3 where available) [31].

Endovascular procedure

MT techniques, first-pass strategy, bridging thrombolysis, rescue manoeuvres, and periprocedural medications were at the operator's discretion within institutional norms. Procedural complications (e.g., distal embolization, perforation, subarachnoid haemorrhage) were captured from operative and immediate post-procedure documentation.

Outcomes

Primary efficacy outcome: Functional outcome at 90 days after stroke, measured by the modified Rankin Scale (mRS) and analysed dichotomously as good outcome (mRS 0-2) versus poor outcome (mRS 3-6). Secondary efficacy outcomes: Angiographic reperfusion quality immediately post-procedure, scored using mTICI; predefined categories: good reperfusion = mTICI 2b-3 versus <2b; where available, an exploratory contrast of near-complete/complete reperfusion (eTICI 2c/3) was examined. Anterior-circulation threshold analysis: discriminatory performance of baseline DWI core volume for predicting poor 90-day outcome, using receiver operating characteristic (ROC)/area under the curve (AUC) with a Youden-optimised cut-point [19,27].

Statistical analysis

Continuous variables were compared between 90-day outcome groups using Welch’s two-sample t-test when normality was plausible or Mann-Whitney U otherwise; categorical variables used χ² test (or Fisher’s exact when expected counts <5). Two-sided α = 0.05 defined statistical significance. For the key continuous comparison (baseline DWI core volume), unequal variances were anticipated a priori; results are reported with t, df, p, mean difference, 95% CI, and Hedges’ g. Multivariable associations with 90-day functional independence (mRS ≤ 2) were estimated by binary logistic regression (reporting odds ratio (OR), 95% CI, and Wald χ²/p), with baseline DWI core entered per 10 mL, and prespecified inclusion of mTICI and sICH. Discrimination was assessed using ROC AUC with the Youden index to identify a pragmatic anterior-circulation core threshold. The study used previously published clinical and imaging scales strictly for scoring; no proprietary questionnaires were administered, and no instrument was reproduced verbatim. As such, no additional permissions were required beyond citation.

## Results

Participant flow and cohort profile

Forty-one consecutive adults with large-vessel occlusion underwent mechanical thrombectomy within 24 hours and completed 90-day follow-up. The mean age was 68.2 ± 9.7 years; 21/41 (51.2%) were men. Screening and flow. CT/CTA-only triage was not used during the study window; all patients who proceeded to thrombectomy underwent pre-procedural MRI-DWI for core estimation. No patient declined thrombectomy for financial reasons. Good angiographic reperfusion (mTICI 2b-3) was achieved in 38/41 (92.7%). Collateral status was graded “good” in 28/41 (68.3%). At 90 days, 21/41 (51.2%) attained functional independence (mRS ≤ 2). sICH occurred in 14/41 (34.1%) (Table 1).

**Table 1:** Cohort characteristics, procedural success, and clinical outcomes (N = 41) *Good collaterals per ASITN/SIR for anterior and BATMAN for posterior circulation, using conventional thresholds. Descriptive only; no inferential tests. Two-sided α = 0.05 elsewhere. mTICI: modified Thrombolysis in Cerebral Infarction, ASITN: American Society of Interventional and Therapeutic Neuroradiology, SIR: Society of Interventional Radiology, BATMAN: Basilar Artery on Computed Tomography Angiography.

Variable	Value
Age, years (mean ± SD)	68.2 ± 9.7
Male sex, n/N (%)	21/41 (51.2)
Good collaterals*, n/N (%)	28/41 (68.3)
Good reperfusion (mTICI 2b-3), n/N (%)	38/41 (92.7)
Functional independence at 90 days (mRS ≤ 2), n/N (%)	21/41 (51.2)
Symptomatic intracranial haemorrhage (sICH), n/N (%)	14/41 (34.1)

Univariable findings

Baseline DWI core volume clearly separated outcomes: patients with good 90-day function had smaller infarcts at presentation than those with poor outcome (39.2 ± 19.3 mL vs 81.6 ± 29.6 mL; p < 0.001). sICH was strongly associated with poor outcome (p < 0.001). In contrast, collateral grade and time-to-treatment metrics did not differ significantly between outcome groups (all p > 0.05) (Table 2).

**Table 2:** Univariable comparison by 90-day outcome (mRS ≤ 2 vs ≥ 3)* *Collateral grading as in Table 1 (p > 0.05), sICH presence vs absence (p < 0.001), and time metrics (workflow intervals; p > 0.05). Group-wise counts are not displayed; significance testing reflects the study dataset. DWI core volume derived via ABC/2. Welch’s t-test for DWI core: t (32.45) = −5.41, p = < 0.001; mean difference −42.4 mL (95% CI −58.4 to −26.4); Hedges’ g = 1.67. Categorical variables analysed with χ² or Fisher’s exact test (two-sided); report χ²(df) and p from the final cross-tab. Significance threshold α = 0.05.

Variable	Good outcome (mRS ≤ 2)	Poor outcome (mRS ≥ 3)	Between-group p
Baseline DWI infarct volume, mL (mean ± SD)	39.2 ± 19.3	81.6 ± 29.6	< 0.001

Multivariable modelling of functional independence

In an adjusted logistic regression with 90-day functional independence (mRS ≤ 2) as the dependent variable, three variables retained independent associations: smaller infarct volume (OR 0.31; 95% CI 0.15-0.67; p = 0.002), higher reperfusion grade (mTICI category; OR 15.8; p = 0.04), and absence of sICH (sICH OR 0.13; p = 0.006; indicating lower odds of independence when sICH is present). Collateral grade and time metrics were not independently associated after adjustment (Table 3).

**Table 3:** Multivariable logistic regression for 90-day functional independence (mRS ≤ 2). Baseline diffusion weighted image (DWI) infarct volume (per 10 mL). mRS: modified Rankin Scale. NR: 95% confidence interval not reported in the summary dataset. Odds ratio (OR) < 1 denotes lower odds of independence; OR > 1 denotes higher odds. Logistic regression (Wald tests): DWI core per 10 mL OR 0.31, Wald χ² = 9.41, p = 0.002;  modified Thrombolysis in Cerebral Infarction (mTICI) OR 15.8, Wald χ² ≈ 4.21, p = 0.04; Symptomatic intracranial haemorrhage (sICH) OR 0.13, Wald χ² ≈ 7.51, p = 0.006; α = 0.05.

Predictor	Adjusted OR (95% CI)	p-value
Baseline DWI infarct volume (continuous)	0.31 (0.15-0.67)	0.002
Reperfusion grade (mTICI category)	15.8 (NR)	0.04
Symptomatic ICH (present vs absent)	0.13 (NR)	0.006

Threshold analysis (anterior circulation)

Restricting to anterior-circulation cases, baseline infarct volume alone showed excellent discrimination for poor outcome (AUC 0.91). The Youden-optimised cut-point was 50 mL, yielding a sensitivity 88% and specificity of 85% for predicting poor 90-day prognosis using the ABC/2-derived DWI core (Table 4).

**Table 4:** ROC analysis for anterior-circulation cases (baseline DWI core predicting poor outcome) ROC: Receiver operating characteristic, area under the curve (AUC) = 0.91; Youden J = 0.73 at 50 mL (sensitivity 88%, specificity 85%); α = 0.05.

Metric	Value
AUC (95% CI)	0.91 (-)
Optimal core cut-point (Youden)	50 mL
Sensitivity at 50 mL	88%
Specificity at 50 mL	85%

Safety signals

Procedure-related sICH was the dominant safety event (34.1%) and was tightly linked to unfavorable 90-day status (p < 0.001). Group comparison. Baseline, imaging, and procedural features stratified by sICH status are presented in Table 5; patients with sICH had larger baseline DWI cores and much lower rates of independence at 90 days, whereas collateral grade and time metrics did not differ materially. No additional complication category independently shifted outcomes once infarct volume, mTICI grade, and sICH were considered.

**Table 5:** Characteristics by sICH status Welch’s t-test for means; Mann–Whitney U for medians (NIHSS, passes, puncture-to-reperfusion); Fisher’s exact/χ² for categorical. Two-sided α=0.05. NIHSS: National Institutes of Health Stroke Scale, DWI: Diffusion-weighted imaging, mTICI: modified Thrombolysis in Cerebral Infarction, IQR: Inter-quartile range, IVT:

Domain	Variable	No sICH (n=27)	sICH (n=14)	p-value
Baseline	Age, years (mean ± SD)	67.5 ± 9.2	69.6 ± 10.1	0.53
	Male sex, n/N (%)	12/27 (44.4)	9/14 (64.3)	0.33
	NIHSS, median (IQR)	14 (10–18)	17 (13–20)	0.09
	Hypertension, n/N (%)	17/27 (63.0)	10/14 (71.4)	0.58
	Diabetes, n/N (%)	9/27 (33.3)	6/14 (42.9)	0.53
	Atrial fibrillation, n/N (%)	3/27 (11.1)	3/14 (21.4)	0.36
	Prior stroke/TIA, n/N (%)	4/27 (14.8)	3/14 (21.4)	0.67
	Smoking/tobacco, n/N (%)	8/27 (29.6)	6/14 (42.9)	0.36
Imaging	DWI core, mL (mean ± SD)	45 ± 20	70 ± 25	0.0038
	Good collaterals, n/N (%)	22/27 (81.5)	6/14 (42.9)	0.017
	Posterior territory, n/N (%)	3/27 (11.1)	2/14 (14.3)	0.73
Procedure	mTICI 2b–3, n/N (%)	26/27 (96.3)	12/14 (85.7)	0.265
	eTICI 2c/3, n/N (%)*	15/27 (55.6)	6/14 (42.9)	0.43
	Passes, median (IQR)	2 (1–3)	3 (2–4)	0.07
	Puncture-to-reperfusion, min, median (IQR)	45 (35–60)	55 (45–70)	0.11
	Bridging IVT, n/N (%)	8/27 (29.6)	6/14 (42.9)	0.36
Outcome	mRS 0–2 at 90 d, n/N (%)	19/27 (70.4)	2/14 (14.3)	0.0009

## Discussion

This prospective cohort shows a simple pattern with real clinical bite: baseline DWI core, the quality of reperfusion, and the avoidance of sICH largely dictate 90-day independence after MT, whereas collateral scores and clock times fade once those three are in the model. Smaller cores independently tracked with better outcomes; higher angiographic grade carried added benefit; sICH strongly penalised recovery. A pragmatic ABC/2-derived threshold of ~50 mL in the anterior circulation offered high discrimination for poor prognosis (AUC 0.91), giving teams a clear, bedside anchor for counselling and audit.

These signals sit squarely within the modern “tissue-first” arc. Trial-only meta-analysis across large-core RCTs has shown higher chances of functional independence with EVT, without a mortality penalty despite increased ICH risk [5]. Contemporary practice standards, aggregating RESCUE-Japan LIMIT, SELECT2, ANGEL-ASPECT, and peers, endorse EVT selection for large cores in early and late windows, while acknowledging safety trade-offs [3]. State-of-the-art reviews echo the same drift: eligibility has widened beyond rigid clocks, and the procedural goal has become maximal reperfusion ideally eTICI 2c/3 because better reperfusion maps to better function [1,2]. Our data align: reperfusion grade retained an independent association with independence at 90 days.

Core size is not just a baseline descriptor; it shapes the ceiling of recovery. Large-registry work suggests treatment effect is not linear across the core spectrum: benefit is evident around 70-100 mL but vanishes and may tilt toward harm at ≥100 mL [6]. Editorial commentary has urged patient-level meta-analyses to test whether “large core” meaningfully modifies EVT benefit or whether current subgroup signals reflect mixed imaging definitions and limited power [8]. Against that backdrop, our 50 mL poor-prognosis cutoff is deliberately conservative. Two practical reasons may explain the lower threshold: first, ABC/2 on DWI can diverge from automated rCBF-based core on CTP, nudging volumes upward or downward depending on lesion geometry; second, our sICH burden was high, which can compress the viable range where good outcomes are plausible. The take-home is calibration, not exclusion: thresholds should flag high-risk trajectories and sharpen consent, not serve as absolute bars to reperfusion, particularly because even patients with extremely low ASPECTS can sometimes return to mRS 0-3 if recanalization succeeds [32].

Collateral physiology deserves a careful reading. Broad syntheses show that better collaterals slow infarct growth, lower haemorrhagic risk, and raise the odds of good outcomes after reperfusion [11]. Large multicentre registries confirm that good angiographic collaterals predict independence and that successful reperfusion helps across collateral strata; the penalty for time is steeper when collaterals are poor [12]. In our adjusted model, collateral grade did not persist once core, mTICI, and sICH were considered. Two interpretations can co-exist: (i) collateral effects are mediated through smaller cores and lower haemorrhage risk; (ii) with aggressive pursuit of high-grade reperfusion, the incremental predictive weight of collateral scores shrinks. Tissue-level collateral surrogates support this view: the hypoperfusion intensity ratio (HIR) modifies the payoff of pushing to eTICI 2c/3 marginal when HIR is low (good collaterals), substantial when HIR is high (poor collaterals) [17]. Practically, this argues for reading collateral biology as a guide to “how hard to push” for near-complete reperfusion rather than as a veto.

Technique debates should be pragmatic. Randomized evidence suggests direct MT yields similar 90-day function to bridging IVT+MT, with trade-offs slightly lower angiographic success but less “any ICH” with direct MT [22]. Collateral-stratified observational data hint that patients with robust collaterals may preferentially benefit from direct MT rather than IVT+EVT, though those signals are single-centre and hypothesis-generating [33]. Our dataset was not powered to adjudicate technique effects, but the independent association of reperfusion grade reinforces a simple operational rule: whatever the route, finish with as complete a reperfusion as safely achievable.

Safety remains the quiet determinant. Our sICH rate (34.1%) was markedly higher than typical RCT and registry ranges (≈4-13%) and unsurprisingly tracked with poor outcomes. Known sICH risk factors include higher NIHSS, prolonged procedural times, diabetes, carotid-T lesions, and early BP variability [20,21]. In a stratified comparison (Table 5), the sICH group presented with larger baseline DWI cores and had markedly lower odds of 90-day independence, while collateral category and workflow times were not materially different between groups. This pattern suggests that haemorrhagic complications likely mediate much of the poor outcome signal rather than reflect systematic differences in collateral status or speed of care. Operationally, this reinforces haemorrhage stewardship tight blood-pressure protocols, minimising passes, and early post-procedure imaging as a high-leverage target alongside pursuing near-complete reperfusion. Given the sample size, these subgroup findings are descriptive and should be validated in larger cohorts. Guidelines suggest post-MT systems that standardize early imaging (≤24 h), codify BP targets, and structure antithrombotic decisions [4]. In practical terms, improving haemorrhage stewardship in our pathway could restore the prognostic value of collaterals and enlarge the envelope for favourable recovery especially around the gray zone of 50-80 mL cores.

Early post-MT imaging can refine prognosis beyond the baseline picture. Independent of initial core, 24-hour DWI lesion size and infarct growth carry strong, inverse associations with 90-day independence; adding these variables improves model performance [25]. This is actionable: routine DWI (or standardized CT-based surrogates where MRI access is limited) at ~24 h can triage monitoring intensity, trigger edema management earlier, and guide realistic family conversations when growth is brisk.

Selection pathways should remain nimble across resource settings. While automated perfusion underpinned late-window trials, collateral-based and NCCT/CTA-based routes can safely broaden access when software would delay care [4,24]. Venous-outflow measures such as COVES on multiphase CTA add predictive power for both excellent reperfusion and outcomes, and are implementable without perfusion software [15]. In public-system contexts, pragmatic RCTs like RESILIENT and PROBE-style large-core trials seek to translate efficacy into policy, with core labs, imaging training, and cost-effectiveness baked in [9,10]. Our findings particularly the weight of reperfusion quality and the sICH penalty, fit these implementation goals and point to where local protocols can move the needle fastest.

How should teams use a 50 mL threshold today? As a risk flag, not a stop sign. The RCT-only meta-analysis in low-ASPECTS ranges supports benefit despite higher haemorrhage risk [34], and guideline syntheses show functional gains across imaging strata, with neutral mortality [3,5]. At the same time, registry data suggest diminishing returns as cores pass ~100 mL [6]. A balanced stance is to proceed when systems can deliver fast, high-grade reperfusion and vigilant post-MT care, while being candid about expected trajectories when cores are already large. This is especially relevant in very old patients: even in nonagenarians, simple MRI collateral surrogates (FVH-ASPECTS) retain prognostic value and can support reperfusion decisions [13].

This work has strengths that help it travel: consecutive real-world enrollment, standardised DWI-based core estimation, and complete 90-day ascertainment. Limitations temper certainty: small sample size; ABC/2 approximations rather than automated segmentation; mixed anterior-posterior occlusions with territory-specific collateral scales; collapsed mTICI categories; and a high sICH rate that may have masked the independent contribution of collateral status. These constraints argue for validation in larger, harmonised cohorts and for sensitivity analyses using automated core estimation and eTICI subgrades. Because selection required MRI-DWI, our cohort may not reflect CT/CTA-only workflows used in some settings, which can limit generalisability.

Three practice implications fall out cleanly. First, measure core volume early and name the risk use ~50 mL as a caution threshold for counselling while avoiding reflex exclusion [3,5,7]. Second, optimise for near-complete reperfusion when feasible, especially in poor tissue-level collaterals; the functional dividend is largest there [1,17]. Third, drain risk from the back end: standardise post-MT BP policies, early imaging, and haemorrhage protocols to reduce sICH and unlock the benefit that reperfusion promises [4,20,21]. Layer on a 24-hour DWI or a CT-based surrogate to personalise monitoring and escalation [25]. Where perfusion software is scarce, lean on NCCT/CTA with collateral and venous-outflow scoring to keep doors open without slowing care [4,15,24].

## Conclusions

Outcomes after mechanical thrombectomy were driven by three levers: the volume of infarcted tissue at baseline, the completeness of reperfusion, and the occurrence of symptomatic intracranial haemorrhage. Once these factors were considered together, collateral grades and clock times added little additional prognostic weight. A DWI-derived core of ~50 mL in the anterior circulation is best used as a risk flag for counselling and audit, not as a gate that blocks treatment.

Operationally, teams should aim for near-complete reperfusion by the safest route and protect that win with disciplined post-procedure care early imaging, firm blood-pressure targets, and careful antithrombotic decisions to limit haemorrhagic complications that otherwise erase gains. However, given the small, single-centre cohort (n=41), the MRI-DWI-only selection pathway, and a relatively high sICH rate, these signals are exploratory and not practice-changing. Before altering local selection thresholds or pathways, they should be externally validated in larger, harmonised cohorts with automated core and eTICI adjudication and standardised post-MT haemorrhage protocols. Until then, the present findings are most useful for quality improvement and service review.
